# Correlation Between Quantitative PSMA PET Parameters and Clinical Risk Factors in Non-Metastatic Primary Prostate Cancer Patients

**DOI:** 10.3389/fonc.2022.879089

**Published:** 2022-04-22

**Authors:** Sebastian Zschaeck, Stephanie Bela Andela, Holger Amthauer, Christian Furth, Julian M. Rogasch, Marcus Beck, Frank Hofheinz, Kai Huang

**Affiliations:** ^1^ Department of Radiation Oncology, Charité – Universitätsmedizin Berlin, Corporate Member of Freie Universität Berlin and Humboldt-Universität zu Berlin, Berlin, Germany; ^2^ BIH Charité Clinician Scientist Program, Berlin Institute of Health at Charité – Universitätsmedizin Berlin, BIH Biomedical Innovation Academy, Berlin, Germany; ^3^ Department of Nuclear Medicine, Charité – Universitätsmedizin Berlin, Corporate Member of Freie Universität Berlin and Humboldt-Universität zu Berlin, Berlin, Germany; ^4^ PET Center, Institute of Radiopharmaceutical Cancer Research, Helmholtz-Zentrum Dresden-Rossendorf, Dresden, Germany

**Keywords:** PSMA, prostate specific membrane antigen, positron emission tomography, primary prostate cancer, quantitative PET parameters

## Abstract

**Background:**

PSMA PET is frequently used for staging of prostate cancer patients. Furthermore, there is increasing interest to use PET information for personalized local treatment approaches in surgery and radiotherapy, especially for focal treatment strategies. However, it is not well established which quantitative imaging parameters show highest correlation with clinical and histological tumor aggressiveness.

**Methods:**

This is a retrospective analysis of 135 consecutive patients with non-metastatic prostate cancer and PSMA PET before any treatment. Clinical risk parameters (PSA values, Gleason score and D’Amico risk group) were correlated with quantitative PET parameters maximum standardized uptake value (SUV_max_), mean SUV (SUV_mean_), tumor asphericity (ASP) and PSMA tumor volume (PSMA-TV).

**Results:**

Most of the investigated imaging parameters were highly correlated with each other (correlation coefficients between 0.20 and 0.95). A low to moderate, however significant, correlation of imaging parameters with PSA values (0.19 to 0.45) and with Gleason scores (0.17 to 0.31) was observed for all parameters except ASP which did not show a significant correlation with Gleason score. Receiver operating characteristics for the detection of D’Amico high-risk patients showed poor to fair sensitivity and specificity for all investigated quantitative PSMA PET parameters (Areas under the curve (AUC) between 0.63 and 0.73). Comparison of AUC between quantitative PET parameters by DeLong test showed significant superiority of SUV_max_ compared to SUV_mean_ for the detection of high-risk patients. None of the investigated imaging parameters significantly outperformed SUV_max_.

**Conclusion:**

Our data confirm prior publications with lower number of patients that reported moderate correlations of PSMA PET parameters with clinical risk factors. With the important limitation that Gleason scores were only biopsy-derived in this study, there is no indication that the investigated additional parameters deliver superior information compared to SUV_max_.

## Introduction

Various studies were able to show that Gallium-68-labelled prostate-specific membrane antigen (PSMA) positron emission tomography (PET) can improve nodal and distant staging of prostate cancer patients (1, 2). An additional benefit of PET imaging is that imaging parameters can be quantified, e.g., by the calculation of standardized uptake values (SUV), PSMA expressing tumor volume (PSMA-TV) and its derivatives. The maximum SUV (SUV_max_) of tumor lesions has been shown to be prognostic for a plethora of diseases and tumor stages and various PET tracers, including the most commonly used tracer [^18^F]fluorodeoxyglucose (FDG) but also less frequently used tracers (3, 4). Recent studies reported that (semi-)quantitative PSMA parameters appear to be a promising prognostic parameter. These investigations were mainly performed in advanced metastatic disease with patients prior to PSMA radioligand treatment (5, 6). In these cohorts of patients, high PSMA uptake seems to be associated with adverse outcome. So far, no data is available for locally confined disease and primary staging of prostate cancer, probably due to the relatively short follow-up time with this novel radiotracer.

Regarding focal radiotherapy treatment escalation in non-metastatic primary prostate cancer patients, an important issue is the potential correlation between quantitative PSMA ligand uptake measures and tumor aggressiveness, e.g. its correlation with the histopathological defined Gleason score. Additional PET parameters could help in the decision for more personalized treatment options like focal radiation boost to tumors, which has shown promising results in magnetic resonance imaging (MRI) guided boost delineation and is currently investigated in PSMA based focal dose escalation trials (7–9). Only weak to moderate correlation has been observed between PSMA PET SUV_max_ during initial staging of prostate cancer and Gleason scores obtained by biopsy. Similar modest correlations were reported for serum PSA values and SUV_max_ (2, 10, 11). Most studies only investigated SUV_max_ and did not analyse further quantitative PET metrics. A novel quantitative PET parameter is tumor asphericity (ASP). ASP is a measure of tumor shape irregularity and has shown a strong association with patient outcome in various diseases and for different PET tracers (12–15). In a recent study with a relatively small number of patients, ASP from [^68^Ga]Ga-PSMA-11 PET was strongly associated with Gleason scores in patients with primary prostate cancer (16).

The aim of our study was to investigate the correlation between different quantitative PSMA parameters, including PSMA derived tumor volume (PSMA-TV) and ASP, with Gleason scores and PSA values and examine if one of these parameters outperforms SUV_max_, especially regarding personalized treatment options of the primary tumour in patients without evidence of loco-regional or distant tumor lesions.

## Patients and Methods

### Patient Cohort

For this retrospective analysis, all patients that underwent [^68^Ga]Ga-PSMA-11 PET/CT imaging between January 2015 and December 2018 at a single tertiary hospital were screened for inclusion and exclusion criteria. Imaging findings and implications for staging of patients that were included until March 2018 have been previously published (17). For the current analysis, all additional consecutive patients with PSMA imaging until end of December 2018 were re-evaluated. Only treatment-naive patients without evidence for lymphonodal or distant metastases were included for further quantitative analyses. Since PSMA PET imaging is not part of the routine staging, referral for imaging was left at the discretion of the referring urologist or radiation oncologist. All except one patient had histologically confirmed prostate-cancer. The remaining patient had steadily rising PSA values during active surveillance, although repeated biopsies only revealed Gleason scores of 4. This patient was diagnosed with prostate cancer based on clinical findings (PSA increase, and characteristic findings in magnetic resonance imaging and PSMA PET/CT) and treated with radiotherapy.

### Clinical Parameters

Clinical data were collected from patient files and electronic databases and included serological prostate-specific antigen (PSA) values, clinical T stage and Gleason scores obtained during biopsy prior to imaging. For a sub-group of patients that underwent surgery after PSMA PET imaging at the same institution, surgical Gleason scores were collected. Gleason scores were grouped following the recommendations of the 2014 International Society of Urological Pathology (ISUP) consensus conference on Gleason grading of prostatic carcinoma (18). Patients were allocated to low, intermediate, or high-risk groups based on the established D’Amico risk classifier (19).

### Image Acquisition

Imaging was performed as previously described (17). Briefly, PSMA PET/CT was performed with the radiotracer [^68^Ga]Ga-PSMA-11-HBED-CC on a dedicated PET/CT scanner (Gemini TF 16; Philips, Netherlands) with Philips Astonish TF technology. [^68^Ga]Ga-PSMA-11-HBED-CC was injected intravenously (median activity: 153 MBq; range: 71-227 MBq). PET imaging was performed after a median time of 98 minutes after injection (range: 39-188 minutes). Patients were placed in supine position and scanned from base of skull to the proximal femora (scan duration: 90 to 180 s per bed position; 3D acquisition mode; bed overlap: 53.3%). Attenuation correction was based on non-enhanced low-dose CT (automatic tube current modulation; maximum tube current-time product: 50 mA; tube voltage: 120 kV; gantry rotation time: 0.5 s) reconstructed with a slice thickness of 5 mm (convolution kernel: B08). PET raw data was reconstructed using iterative reconstruction with TOF analysis (Philips Astonish TF technology; BLOB-OS-TF; iterations: 3; subsets: 33). The projection data was reconstructed with 4 mm slice thickness (voxel size: 4×4×4 mm^3^) (17).

### Image Evaluation

In a first step, a large spheric mask was placed around the prostate and base of seminal vesicles. The PSMA expressing part of the primary tumor was delineated inside this mask based on a threshold of 41% SUV_max_ as suggested by a recent analysis (20). The resulting volumes of interest (VOI) were inspected visually by an experienced observer (SZ), and tracer uptake of surrounding normal tissue (bladder and/or rectum) was manually excluded. Patients who exhibited only low or diffuse tracer accumulation in the respective lesion were manually delineated by selecting the most intense single voxel, the volume in these patients was regarded 0.1 ml. This was the case in four patients.

For the obtained VOIs, ASP was computed according to the following formula, where V is the volume of the VOI and S is its surface.


$$\text{ASP}=\sqrt[{3}]{\frac{1}{36\pi }\frac{S^{3}}{V^{2}}-1}$$

ASP is equal to zero for spheres. For non-spherical shapes ASP is higher than 0 and is a quantitative measure of the degree of deviation from a spherical shape.

In addition, the PSMA based tumor volume (PSMA-TV), the maximum standardized uptake value (SUV_max_) and average standardized uptake value (SUV_mean_) and SUV_peak_ were calculated. SUVs were computed using the patients body weight. All VOI definitions and image analyses were performed using the ROVER software, version 3.0.41 (ABX, Radeberg, Germany).

### Statistical Analyses

**Figure 1:**
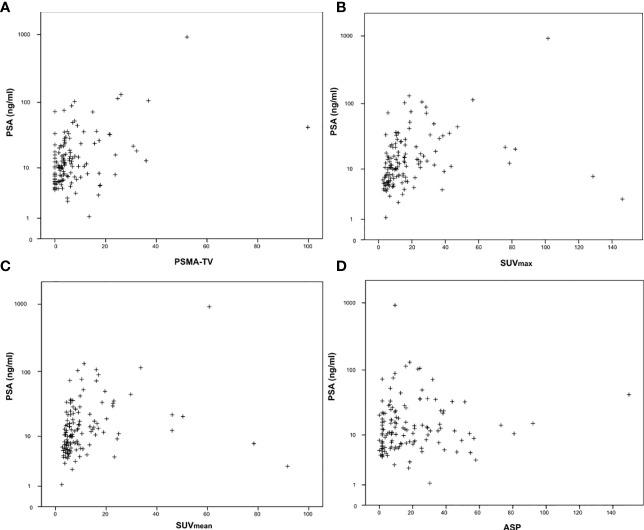
Correlation between serum prostate-specific antigen (PSA) values and quantitative PSMA-PET parameters. **(A)** PSMA-derived tumor volume (PSMA-TV), **(B)** Maximum standardized uptake value (SUVmax), **(C)** Mean standardized uptake value (SUVmean) and **(D)** Tumor asphericity (ASP). PSA values are plotted on a logarithmic scale.

**Figure 2:**
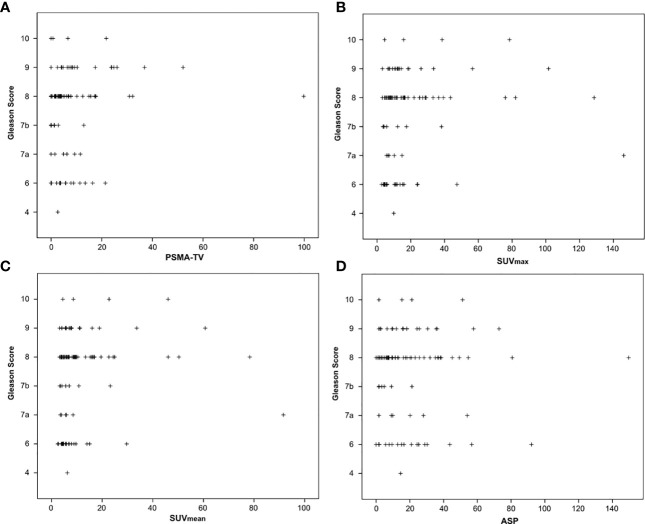
Correlation between Gleason scores obtained by biopsy before imaging and quantitative PSMA-PET parameters. **(A)** PSMA-derived tumor volume (PSMA-TV), **(B)** Maximum standardized uptake value (SUVmax), **(C)** Mean standardized uptake value (SUVmean) and **(D)** Tumor asphericity (ASP).

The nonparametric Spearman correlation was used for calculation of correlations between imaging and clinical parameters to avoid bias due to existing outliers (as depicted in **Figures 1**, **2**). Receiver operating characteristics (ROC) curves were plotted to show sensitivity and specificity of each quantitative PET parameter for detection of high-risk prostate cancer (as defined by D’Amico criteria). Area under the curve (AUC) comparison between quantitative PET parameters were calculated using the DeLong test (MedCalc version 19.3, MedCalc Software Lt, Ostend, Belgium). All other statistical calculations and figure plots were performed using SPSS version 24 (IBM Corporation, Armonk, NY, USA).

## Results

Most patients had high-risk prostate cancer. **Table 1** summarizes clinical characteristics and quantitative imaging findings of the study cohort.

**Table 1:** Patient and PSMA-PET tumor characteristics.

Median age (range)	72 years (49 – 88 years)
**Median PSA** (range)	11.4 (1.1 – 920)
**Gleason Score** (biopsy)	
n/a	27 (20%)
≤ 6	24 (18%)
7a	6 (4%)
7b	7 (5%)
8	48 (36%)
9	19 (14%)
10	4 (3%)
**Clinical T stage**	
n/a	41 (30%)
1	57 (42%)
2	25 (19%)
3	9 (7%)
4	3 (2%)
**D’Amico risk group**	
n/a	22 (16%)
Low-risk	8 (6%)
Intermediate-risk	19 (14%)
High-risk	86 (64%)
**Gleason Score** (surgery)	
≤ 6	1 (3%)
7a	9 (27.5%)
7b	11 (33.5%)
8	4 (12%)
9	7 (21%)
10	1 (3%)
**Median PSMA-TV** (range)	3.8 ml (0 – 99.8 ml)
**Median SUV_max_** (range)	11.0 (2.7 – 146.0)
**Median SUV_mean_** (range)	6.4 (2.5 – 91.6)
**Median ASP** (range)	9.8 (0 – 149.7)

The investigated quantitative PSMA PET parameters were significantly inter-correlated with correlation coefficients between 0.20 and 0.95. The only exception was SUV_mean_ and ASP, which were not significantly correlated (p = 0.79). Details are shown in **Supplementary Table 1**. Regarding correlation between quantitative parameters of the primary tumor and clinical parameters, a significant, however low to moderate correlation with initial serum PSA values (Spearman rho between 0.19 and 0.45, all p < 0.05; **Table 2**; **Figure 1**) was observed. Correlation with Gleason scores obtained by previous biopsy was slightly lower (Spearman rho between 0.17 and 0.31, all p < 0.05 except for ASP; **Figure 2**).

**Table 2:** Correlation between initial PSA values and biopsy-derived Gleason scores with quantitative PSMA-PET parameters.

	PSMA-TV	SUV_max_	SUV_mean_	ASP
**PSA**	r = 0.366	r = 0.450	r = 0.442	r = 0.188
	p < 0.001	p < 0.001	p < 0.001	p = 0.031
	(n = 132)	(n = 131)	(n = 131)	(n = 132)
**Gleason**	r = 0.306	r = 0.307	r = 0.233	r = 0.171
	p = 0.001	p = 0.001	p = 0.016	p = 0.076
	(n = 108)	(n = 107)	(n = 107)	(n = 108)

**Figure 3:**
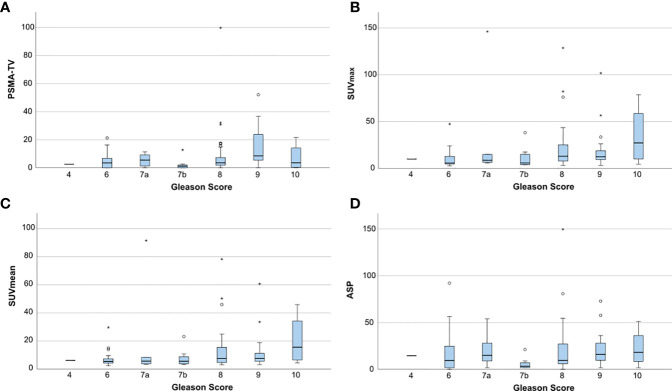
Boxplots showing the distribution of quantitative PET parameters for each Gleason score. **(A)** PSMA-derived tumor volume (PSMA-TV), **(B)** Maximum standardized uptake value (SUVmax), **(C)** Mean standardized uptake value (SUVmean) and **(D)** Tumor asphericity (ASP). Outliers are plotted as points (< 3 * interquartile range) or asterisks (> 3 * interquartile range).

**Figure 3** shows the distribution of quantitative PET parameters for each Gleason score.

**Figure 4:**
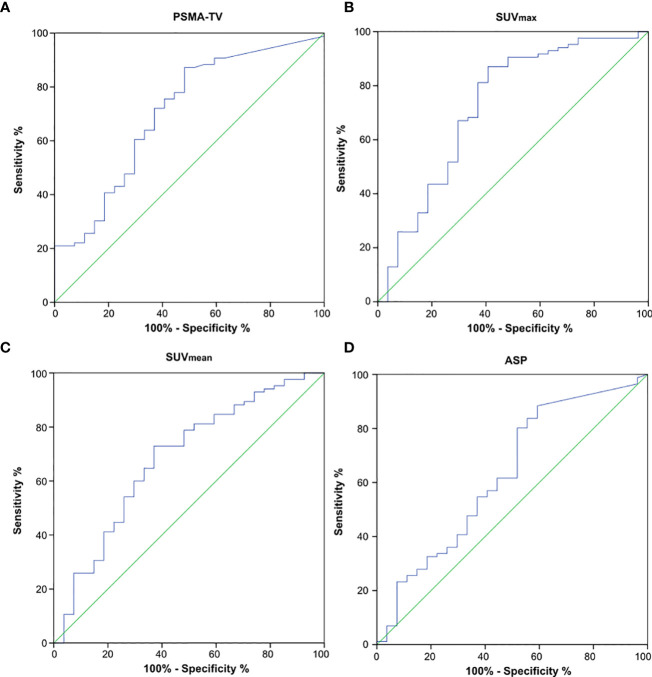
Receiver operating characteristics (ROC) curves to detect high-risk prostate cancer using quantitative PSMA-PET parameters. **(A)** PSMA-derived tumor volume (PSMA-TV), **(B)** Maximum standardized uptake value (SUVmax), **(C)** Mean standardized uptake value (SUVmean) and **(D)** Tumor asphericity (ASP).

AUC analysis regarding the differentiation of high-risk from low- or intermediate-risk prostate cancer patients revealed poor to fair sensitivity and specificity for all investigated imaging parameters. AUC plots are depicted in **Figure 4** and the respective values are shown in **Table 3**. Comparison between AUC characteristics for different PET parameters showed that SUV_max_ is significantly better suited than SUV_mean_ to predict high-risk prostate cancer (p = 0.035), no significant differences between other quantitative metrics could be observed as shown in **Table 4**. Additionally, SUVpeak was investigated in the whole cohort, SUVpeak showed a very high correlation with SUVmax (r = 0.99, p < 0.001) and similar results regarding all investigated endpoints as shown in **Supplementary Figure 1**.

**Table 3:** Area under the curve (AUC) characteristics for the investigated quantitative PSMA-PET parameters to detect high-risk prostate cancer.

	AUC	95% confidence interval
**PSMA-TV**	0.70	0.61 – 0.79
**SUV_max_**	0.73	0.63 – 0.81
**SUV_mean_**	0.68	0.59 - 0.77
**ASP**	0.63	0.53 - 0.72

**Table 4:** Comparison of AUC characteristics to detect high-risk prostate cancer using the DeLong test.

	PSMA-TV	SUV_max_	SUV_mean_	ASP
**PSMA-TV**	–	difference: 0.023	difference: 0.020	difference: 0.073
		p = 0.74	p = 0.80	p = 0.051
**SUV_max_**	–	–	difference: 0.043	difference: 0.096
			p = 0.035	p = 0.22
**SUV_mean_**	–	–	–	difference: 0.053
				p = 0.54

Since Gleason scores obtained from biopsy might over- or underestimate surgically obtained Gleason scores of whole prostate specimens, a sub-group of 38 surgically treated patients was further evaluated. Similar correlation coefficients as in the main analysis (but with each p>0.05) were obtained between quantitative imaging parameters and surgical Gleason scores (**Supplementary Table 2**, **Supplementary Figure 2**).

## Discussion

PSMA PET has shown great potential for focal treatment strategies. Bettermann and colleagues were able to show that PSMA PET-based tumor delineation is superior to MRI regarding the sensitivity to detect prostate cancer foci on whole mount histopathology specimens (21). Several studies are currently investigating focal treatment escalation by the implementation of PET imaging. Identification of the optimal imaging parameter as a surrogate for tumor aggressiveness is therefore an important need.

In this study, we examined the correlation between PET parameters and clinical risk factors in non-metastatic primary prostate cancer patients. We were able to validate prior publications that reported a moderate correlation between clinical risk parameters like Gleason score, PSA levels or D’Amico risk category and SUV_max_ of primary prostate tumors. Further analysis of additional quantitative PET parameters like ASP or PSMA-TV did not show superiority compared to SUV_max_ in this monocenter investigation. Only a moderate correlation of any investigated parameter with Gleason scores could be observed.

The reported correlation coefficients in our study are comparable with published data on correlations between SUV_max_ and Gleason scores that ranged between 0.096 and 0.5 and correlation coefficients between SUV_max_ and PSA values that ranged between 0.071 and 0.57 (2, 10, 11, 22–27). All but one of these studies reported lower numbers of patients, **Supplementary Table 3** gives an overview of the published data on correlation coefficients.

Gleason scores of needle biopsies show discrepancies with surgical Gleason scores in up to 50% of cases, especially upgrading to higher Gleason scores is a frequent observation (28, 29). This can influence the observed correlations with quantitative PSMA metrics, probably underestimating the real Gleason score. Analysis of the patient sub-group that underwent surgery did not show any significant correlation between the investigated quantitative PET metrics and surgical Gleason grades. However, this is most likely due to the comparatively low number of patients in this sub-group, because correlation coefficients were similar to the correlation coefficients for biopsy-based Gleason scores.

Data on the correlation between quantitative PSMA PET metrics other than SUV_max_ and clinical risk factors are sparse. Meißner and colleagues reported a strong correlation between ASP and Gleason scores (rho 0.88) and a moderate correlation between tumor volume and Gleason scores (rho 0.51) in a small cohort of 37 patients (16). However, patients with lymphatic or distant metastases were not excluded in their analysis, the exact number of patients with extraprostatic lesions was unfortunately not reported. Hoberück et al. evaluated various quantitative PSMA PET metrics including SUV_max_, SUV_mean_ and PSMA-TV. In a small cohort of 21 patients with consecutive PSMA scans before and during androgen deprivation therapy, they observed a strong correlation between the investigated PET parameters and no superiority of a specific parameter (30). The same quantitative parameters were investigated by Schmidkonz et al. in patients with bone metastases. They reported that all quantitative metrics were higher for Gleason scores > 7, but did not provide further comparative details (31).

Our study has several limitations. First, the retrospective nature of the investigation with its known limitations. Second, no spatial correlation analyses with whole-mount histology was performed in surgically resected patients. Current analyses in this regard showed an excellent correlation of PET parameters with intraprostatic tumor foci (32, 33). Third, the used radiotracer might not be the best modality for local tumor assessment. The high urinary clearance of [^68^Ga]Ga-PSMA-11 hampers automatic delineation in close vicinity to the bladder. The necessary manual modifications are observer-dependent and might complicate independent reproducibility. Furthermore, high bladder uptake can potentially affect quantitative PET metrics of the prostate, e.g. by halo artifacts (34). The F-18-labeled PSMA-1007 radiotracer might be superior for evaluation of primary prostate cancer due to its favorable biodistribution, in particular lower bladder activity (35). Furthermore, SUV_max_ in primary prostate cancer lesions are systematically higher with [18F]F-PSMA-1007 compared to [68Ga]Ga-PSMA-11 (36). Nonetheless, a current meta-analysis was not able to show clear superiority of one of the specific PSMA radioligands in the recurrent situation (37). If prolonged uptake times are encountered in routine clinical care, [18F]F-PSMA-1007 could be advantageous over [68Ga]Ga-PSMA-11 by providing beneficial count statistics due to its longer physical half-life. Additionally, the higher positron range of Gallium-68 compared to Fluor-18 results in decreased spatial resolution, although Soderlund et al. observed only marginal differences using clinical PET scanners (38). The range of uptake times in the current analysis was relatively high, which might hamper inter-patient comparability of SUV. Lesion uptake of [68Ga]Ga-PSMA-11 increases over time after injection and has been described as approximately irreversible (39). However, the average increase in lesion SUV between 1h and 3h post injection has been reported to be moderate (25%) (40). The same PET scanner was used in all patients, which benefits comparability of PET parameters between patients. However, strictly speaking, applicability to other scanner models with different image properties and reconstruction methods would require dedicated analyses.

An important strength of our analysis is the restriction to patients without evidence of metastases by imaging including PSMA PET. Inclusion of metastatic patients might partly explain the high heterogeneity between previous publications, especially regarding correlation coefficients with PSA values (which is highly correlated with the total tumor volume). Additionally image evaluation was performed in a standardized fashion and with the observer being blinded to clinical risk parameters.

Overall, the observed association of the investigated quantitative imaging parameters with clinical risk factors is only fair. Novel methods like radiomics might be more suitable to detect high-risk sub-volumes within the prostate (41, 42).

In summary, this comprehensive analysis of quantitative PSMA PET metrics confirms prior studies that showed a moderate correlation with clinical risk factors. All investigated quantitative PET metrics intercorrelated and showed similar association with Gleason score, PSA values or D’Amico risk groups. The widely used reporting of SUV_max_ only seems therefore reasonable for personalized treatment options like focal boost in primary prostate cancer. Further prospective studies in a large cohort are needed to confirm our results, especially regarding the outcome after PET-guided personalized treatment.

## Data Availability Statement

The raw data supporting the conclusions of this article will be made available by the authors, without undue reservation.

## Ethics Statement

The studies involving human participants were reviewed and approved by Ethikkomission der Charité Universitätsmedizin Berlin, Germany. The ethics committee waived the requirement of written informed consent for participation.

## Author Contributions

Study conception and design: SZ and KH. Drafting of manuscript: SZ and SA. Image processing and analysis: SZ, FH, and JR. Study Investigators: SZ, SA, HA, CF, JR, MB, FH, and KH. Interpretation of data: all authors. All authors contributed to the article and approved the submitted version.

## Conflict of Interest

HA declares research grants, travel grants, and lecture fees from Sirtex Medical Europe; HA confirms that none of the above funding sources were involved in the preparation of this paper.

The remaining authors declare that the research was conducted in the absence of any commercial or financial relationships that could be construed as a potential conflict of interest.

## Publisher’s Note

All claims expressed in this article are solely those of the authors and do not necessarily represent those of their affiliated organizations, or those of the publisher, the editors and the reviewers. Any product that may be evaluated in this article, or claim that may be made by its manufacturer, is not guaranteed or endorsed by the publisher.
