# Supplementary material for: Correlation Between Quantitative PSMA PET Parameters and Clinical Risk Factors in Non-Metastatic Primary Prostate Cancer Patients
Source: Front Oncol. 2022 Apr 22;12:879089. doi: 10.3389/fonc.2022.879089 (PMC9074726; doi:10.3389/fonc.2022.879089)
Supplement: Supplementary file 1 [file DataSheet_1.docx]

Supplementary Material

# Supplementary table 1

**Supplementary table 1:** Intercorrelation between quantitative PET parameters

|  | PSMA-TV | SUV_max_ | SUV_mean_ | ASP |
| --- | --- | --- | --- | --- |
| PSMA-TV |  | r = 0.42  p < 0.001 | r = 0.20  p = 0.02 | r = 0.74  p < 0.001 |
| SUV_max_ | r = 0.42  p < 0.001 |  | r = 0.95  p < 0.001 | r = 0.24  p = 0.006 |
| SUV_mean_ | r = 0.20  p = 0.02 | r = 0.95  p < 0.001 |  | r = 0.024  p = 0.79 |

# Supplementary table 2

**Supplementary table 2:** Correlation between surgical Gleason scores and quantitative PET parameters

|  | PSMA-TV | SUV_max_ | SUV_mean_ | ASP |
| --- | --- | --- | --- | --- |
| Surgical  Gleason Score | r = 0.13  p = 0.43 | r = 0.25  p = 0.14 | r = 0.17  p = 0.26 | r = 0.01  p = 0.56 |

# Supplementary table 3: Overview of publications investigating quantitative PSMA-PET metrics and PSA values and/ or Gleason scores.

| **Publication** | **n =** | **Location** | **Correlation Gleason score with SUV_max_** | **Median SUV_max_ Gleason score 6** | **Median SUV_max_ Gleason score 7A** | **Median SUV_max_ Gleason score 7B** | **Median SUV_max_ Gleason score 8** | **Correlation PSA with SUV_max_** |
| --- | --- | --- | --- | --- | --- | --- | --- | --- |
| Hong et al. 2020 | 101 | Wenzhou, China | r = 0.496  p < 0.001 | 5.35 | 8.70 | 11.60 | 18.08 | r = 0.561  p < 0.001 |
| Cytawa et al. 2020 | 70 | Perth, Australia | r = 0.35  p = 0.001 | 9 | 15 | | 22 | r = 0.51  p < 0.001 |
| Uprimny et al. 2017 | 90 | Innsbruck, Austria |  | 5.9 | 8.3 | 8.2 | 21.2 | r = 0.506  p < 0.001 |
| Demirci et al. 2019 | 141 | Istanbul, Turkey | r = 0.50  p<0.001 | 6.4 | 6.02 | 15.58 | 14.2 |  |
| Liu et al. 2018 | 50 | Beijing, China | r = 0.42  p<0.01 | 10.5 | | | 14.2 | r = 0.43  p < 0.01 |
| Eiber et al. 2016 | 53 | Munich, Germany | r = 0.096  p = n.s. |  |  |  |  | r = 0.071  p = n.s. |
| Jena et al. 2018 | 63 | Delhi, India | r = 0.30  p = 0.05 |  | 14.08 | | 18.22 | r = 0.51  p < 0.01 |
| Sachpekidis et al. 2016 | 24 | Heidelberg, Germany | r = 0.28  P < 0.05 |  |  | |  | r = 0.57  p < 0.0001 |
| Donato et al. 2019 | 58 | Brisbane, Australia |  | 3.85 | 6.69 | 9.72 | 8.28 |  |

# Supplementary Figure 1

**Supplementary figure 1:** Correlation between serum PSA values (A), Gleason scores (B and C) and SUV_peak_ and AUC curve for SUV_peak_ (D).

# Supplementary Figure 2


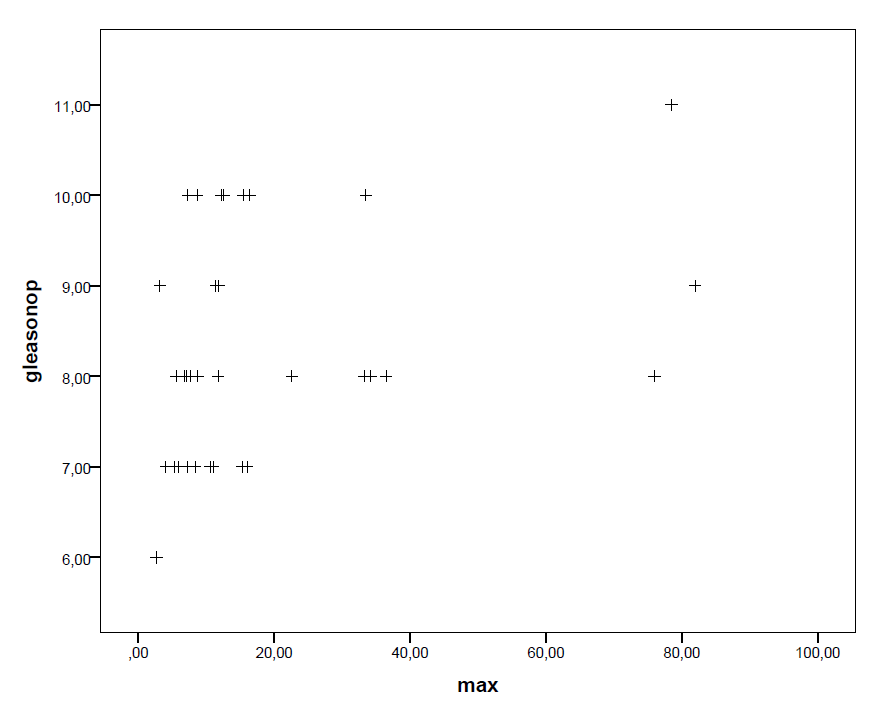


**Supplementary figure 2:** Correlation between surgical Gleason scores and quantitative PSMA-PET parameters for SUV_max_.
